# Frequency and Outcome of Pregnant Females Presenting With Thrombocytopenia at a Tertiary Care Hospital

**DOI:** 10.7759/cureus.49466

**Published:** 2023-11-26

**Authors:** Hira Mumtaz, Raheela Danish, Tayiba Yousaf, Shazia Sehgal, Aiman Jawad, Syed M Ali Haider

**Affiliations:** 1 Gynaecology and Obstetrics, Basildon University Hospital, National Health Service (NHS) Trust, Basildon, GBR; 2 Obstetrics and Gynecology, Jinnah Hospital, Lahore, PAK; 3 Obstetrics and Gynecology, Salma Khalil Clinic, Narowal, PAK; 4 Obstetrics and Gynecology, Fatima Jinnah Medical University, Lahore, PAK; 5 Medicine, Rehman Hospital, Faisalabad, PAK; 6 Medicine, Heavy Industries Taxila (HIT) Hospital, Taxila, PAK

**Keywords:** thrombocytopenia, preterm, placental abruption, parity, blood transfusion, apgar score

## Abstract

Background: Platelet-related problems are more frequently discovered in women during pregnancy because screening is carried out as part of the initial clinic examination using automated blood counts. This study was done to find out the frequency and outcomes of pregnant females presenting with thrombocytopenia at a tertiary care hospital.

Methodology: This cross-sectional study was conducted at the Department of Obstetrics and Gynecology, Jinnah Hospital, Lahore, Pakistan, from April 2023 to September 2023. This study involved 280 pregnant women presenting in the third trimester. Blood examination was acquired, and a platelet count less than 150x10^9^/L was labeled as thrombocytopenia. Outcome variables were frequency of thrombocytopenia, while post-delivery, frequency of placental abruption, preterm delivery, stillbirth, need for blood transfusion, and poor Apgar score were noted and compared among women with and without thrombocytopenia.

Results: In a total of 280 pregnant females, the mean age and gestational age at the time of presentation were 29.34±4.38 years and 31.30±2.87 weeks, respectively. The mean BMI of the females was 27.97±4.72 kg/m^2^. Thrombocytopenia was noted in 34 females (12.1%). Placental abruption, preterm delivery, need for blood transfusion, stillbirth, and poor Apgar score were observed in 1.4%, 4.3%, 8.2%, 1.1%, and 2.1% cases, respectively. Placental abruption (11.8% vs. 0.0%; p<0.001), preterm delivery (29.4% vs. 0.8%; p<0.001), need of blood transfusion (35.3% vs. 4.5%; p<0.001), stillbirth (8.8% vs. 0.0%; p<0.001), and poor Apgar score (17.6% vs. 0.0%; p<0.001) were all significantly higher among pregnant women with thrombocytopenia as compared to those with a normal platelet count.

Conclusion: The frequency of thrombocytopenia was 12.1% among pregnant females. The frequency of placental abruption, preterm delivery, need for blood transfusion, stillbirth, and poor Apgar score were all significantly higher among pregnant women with thrombocytopenia as compared to those with a normal platelet count, irrespective of the patient’s age, parity, and BMI.

## Introduction

Platelet-related problems are more frequently discovered in women during pregnancy because screening is carried out as part of the initial clinic examination using automated blood counts [1,2]. Understanding the various causes of thrombocytopenia during pregnancy makes it easier to diagnose and treat thrombocytopenia in pregnant women [3].

The majority of thrombocytopenia etiologies are caused by gestational thrombocytopenia (>75%), which does not need to be investigated or treated in any particular way. It often develops in the final trimester of pregnancy and corrects itself on its own after birth [4,5]. Additionally, as therapeutic techniques used to treat thrombocytopenic illnesses in pregnant women may contain toxins particular to pregnancy, management strategies require careful considerations [6].

When fetal or neonatal thrombocytopenia occurs, it frequently originates from concurrent neonatal alloimmune thrombocytopenia and is not more common in the offspring of such individuals than it is in non-thrombocytopenic women [7,8]. Women with severe thrombocytopenia have higher odds of composite complications and composite obstetric problems [9]. Some researchers have shown that the frequency of gestational thrombocytopenia is as high as 49.8% [10]. Moreover, a significantly higher frequency of placental abruption was observed in thrombocytopenic females (8.5%), preterm birth (25.6%), stillbirth (6.5%), poor Apgar score (16.8%), and blood transfusion (16.6%) as compared to females with a normal platelet count (p<0.05) [10].

The objective of this study was to determine the frequency and outcomes of pregnant females presenting with thrombocytopenia at a tertiary care hospital. Literature has reported that females with gestational thrombocytopenia have a significantly high risk for adverse obstetrical outcomes, but there have been contradictory statements as well. Therefore, this study aimed to examine the outcome of thrombocytopenia. If a high proportion of females are detected with gestational thrombocytopenia, they might be managed on an early basis so that these preventable complications can be prevented.

## Materials and methods

This descriptive case series study was performed at the Obstetrics and Gynecology Unit III, Jinnah Hospital, Lahore, Pakistan, from April 2023 to September 2023. A sample size of 280 patients was calculated, taking the expected percentage of gestational thrombocytopenia in females presenting for delivery as 7% [1], with a confidence level of 95% and a margin of error of 3%. Sample selection was made employing a non-probability, consecutive sampling technique.

Inclusion criteria were females of the 20-40 age group with a parity <5 and singleton pregnancy confirmed by ultrasonography (USG) presenting for delivery. Exclusion criteria were females with pregnancy-induced hypertension (PIH; BP>140/90 mmHg), preeclampsia (PIH with +1 proteinuria on dipstick), eclampsia (convulsions with PIH), or diabetes (glucose tolerance test (GTT)>140 g/dL). Females with Hb<11 g/dL were also excluded. The objective of the study and the safety issues of the women involved were highlighted to all participants. They were assured that their information would be kept confidential, and informed consent and written consent were obtained from them. An approval from the institutional ethical committee was also obtained (ERB144/13/S1ERB).

After the patients were enrolled, demographic details such as age, parity, and gestational age were recorded. A general physical examination and abdominal examination for the growth and health of the fetus were also noted. Blood samples were obtained using a 5 cc BD syringe (Becton, Dickinson and Company, Franklin Lakes, NJ, United States). Blood was stored in blood storage tubes containing Ringer’s solution. All samples were sent to the institutional laboratory for the assessment of platelet counts. Based on the platelet count reports, females were labeled as having gestational thrombocytopenia or normal. Gestational thrombocytopenia was labeled as a platelet count <150xl0^9^/L. Any patient found to have a platelet count <50x10^9^/L had transfused platelets.

If the placenta separated from the inner wall of the uterus before delivery, as confirmed on USG and clinically (tense, tender abdomen, and paravaginal bleeding), this was considered placental abruption, and labor was monitored as per protocol for high-risk pregnancies. If delivery occurred before 37 weeks (on last menstrual period (LMP)/first-trimester scan), it was considered a preterm delivery. Blood loss was measured during delivery, and Hb estimation was done 24 hours after delivery. Patients received blood transfusion if, at the time of delivery, blood loss was greater than 500 mL, measured by blood in a kidney tray and soaked gauze (weight of wet gauze minus weight of dry gauze, where 1 g=1 mL), or after delivery, Hb<10 g/dL was measured. After delivery, the Apgar score of the baby was noted and labeled as poor if it was less than 7 at one minute. Outcomes in terms of complications in both the mother and fetus were measured. A special, predesigned proforma was used to write down the study information.

Statistical analysis was carried out using IBM SPSS Statistics for Windows, Version 26.0 (Released 2019; IBM Corp., Armonk, New York, United States). Quantitative variables such as age and gestational age were presented by calculating the mean and standard deviation (SD). The qualitative variables such as parity, gestational thrombocytopenia, and outcomes such as placental abruption, preterm delivery, need for blood transfusion, stillbirth (occurrence of a delivery of a dead fetus), and poor Apgar score were expressed as frequency and percentage. The frequency and outcomes of gestational thrombocytopenia were determined. The effect modifiers such as age, parity, and BMI were controlled by making stratified tables and comparing these with respect to thrombocytopenia and outcomes using the chi-square test, considering p<0.05 as statistically significant.

## Results

In a total of 280 women, the mean age was 29.34±4.38 years (ranging between 20 and 40 years). The mean gestational age, parity, and BMI at the time of enrollment were 31.3±2.87 weeks, 2.11±1.05, and 27.97±4.72 kg/m^2^, respectively. There were 112 (40.0%) females who were multiparas (Table 1).

**Table 1:** Baseline characteristics of the study population (n=280).

Characteristics		Number (%)
Age (years)	20-30	175 (62.5%)
	31-40	105 (37.5%)
Parity	Primiparas	111 (39.6%)
	Gravida 2	57 (20.4%)
	Multiparas	112 (40.0%)
BMI (kg/m^2^)	20-25	98 (35.0%)
	25-30	65 (23.2%)
	30-35	117 (41.8%)

At the time of enrollment, thrombocytopenia was documented in 34 women (12.1%). There was no significant association of thrombocytopenia with respect to age (p=0.637), parity (p=0.344), and BMI (p=0.799) (Table 2).

**Table 2:** Frequency of thrombocytopenia with respect to study variables (n=280).

Characteristics		Thrombocytopenia (%age)		p-value
		Yes	No	
Age (years)	20-30	20 (11.4%)	155 (88.6%)	0.637
	31-40	14 (13.3%)	91 (86.7%)	
Parity	Primiparas	17 (15.3%)	94 (84.7%)	0.344
	Gravida 2	7 (12.3%)	50 (87.7%)	
	Multiparas	10 (8.9%)	102 (91.1%)	
BMI (kg/m^2^)	20-25	11 (11.2%)	87 (88.8%)	0.799
	25-30	7 ( (10.8%)	58 (89.2%)	
	30-35	16 (13.7%)	101 (86.3%)	

The frequency of placental abruption, preterm delivery, stillbirth, need for transfusion, and poor Apgar score were noted in four (1.4%), 12 (4.3%), three (1.1%), 23 (8.2%), and six (2.1%) cases, respectively. A significant association of thrombocytopenia was found with placental abruption (p<0.001), preterm delivery (p<0.001), stillbirth (p<0.001), need for transfusion (p<0.001), and poor Apgar score (p<0.001) (Table 3).

**Table 3:** Frequency of outcome measures across thrombocytopenia status (n=280).

Outcome measures		Thrombocytopenia		p-value
		Yes (n=34)	No (n=246)	
Placental abruption	Yes	4 (11.8%)	-	<0.001
	No	30 (88.2%)	246 (100%)	
Preterm delivery	Yes	10 (29.4%)	2 (0.8%)	<0.001
	No	24 (70.6%)	244 (99.2%)	
Stillbirth	Yes	3 (8.8%)	-	<0.001
	No	31 (91.2%)	246 (100%)	
Need for transfusion	Yes	12 (35.3%)	11 (4.5%)	<0.001
	No	22 (64.7%)	235 (95.5%)	
Poor Apgar	Yes	6 (17.6%)	-	<0.001
	No	28 (82.4%)	246 (100%)	

## Discussion

In this study, the frequency of thrombocytopenia was documented in 12.1% of cases. The maternal platelet count declines physiologically by around 10% during pregnancy, while the resulting platelet count at term is typically kept within the normal range. Population-based studies have revealed that the prevalence of gestational thrombocytopenia, which is indicated by a platelet count of fewer than 150x10^9^/L, ranges between 5 and 12% [11,12]. It has been demonstrated that the platelet count increases on days 2-5 postpartum [12]. The causes of the decrease in platelet count are unknown, but they may include hemodilution and faster platelet clearance. Pregnant individuals have few options for directly measuring platelet lifespan, and earlier investigations have not consistently shown decreased platelet survival in pregnancy [12]. The fact that platelet volume has increased while platelet counts have decreased in the third trimester may be a sign of accelerated platelet degradation [13]. Additionally, it is less certain if increased platelet activation occurs in a typical pregnancy, despite the fact that it has been shown to occur in pregnancies affected by hypertension or preeclampsia. Numerous studies have shown that normotensive pregnant women have spontaneous platelet aggregation, enhanced platelet responsiveness to arachidonic acid, and, more recently, increased platelet activation and adhesion [13].

In a prospective study, 21% of the children born from mothers with gestational thrombocytopenia had low platelet counts in their cord blood, although none of the infants experienced sepsis. None of the neonates needed treatment, despite having platelet counts that ranged from 58 to 144x10^9^/L. The authors described that even in cases with proven gestational thrombocytopenia, the platelet count in cord blood should be examined for every child born to a mother with pregnancy-associated thrombocytopenia [14]. Although gestational thrombocytopenia is a benign illness, it is recommended to monitor platelet counts during pregnancy in order to spot any precipitous decline that could lead to hemorrhagic symptoms later [15].

Patients’ mean age in this study was 29.34±4.38 years. Nazli et al. reported a similar mean age of 29.54±0.68 years among patients presenting at Khyber Teaching Hospital, Peshawar, Pakistan [16]. Elveđi-Gašparović et al. reported a similar mean age of 30.8±5.59 years among pregnant women with thrombocytopenia [17]. The mean age described by Elveđi-Gašparović et al. was also similar to ours at 29.8±4.97 years in Croatia, while Habas et al. mentioned it to be 32.56±1.5 years among Libyan women [17,18]. A similar mean age of 28.9±7.17 years and 29.84±6.03 years was claimed by Yassaee et al. and Hussein and El Sawaf among Iranian and Egyptian women, respectively [19,20]. A relatively younger mean age of 24.48±3.62 years has been reported by Dwivedi et al. in the Indian pregnant population with thrombocytopenia [21]. We observed a mean gestational age of 31.30±2.87 weeks among such women. Our study findings coincide with those of Nazli et al., whose conclusion for the mean gestational age was 31.39±0.48 weeks in the local pregnant population having thrombocytopenia [16], and Brohi et al., who reported a mean gestational age of 33.26±5.9 weeks among such women in another local study [22]. We observed a mean BMI of 27.97±4.72 kg/m^2^ among such patients. A similar mean BMI of 29.41±1.04 kg/m^2^ has been reported by Nazli et al. in the local population [16]. However, Chauhan et al. reported it to be 20.95±2.09 kg/m^2^ in Indian patients [23].

In the present study, there were 39.6% primiparas and 40.0% multiparas. Lin et al. reported that 41.4% of Chinese women were primiparas [24]. Elveđi-Gašparović et al. stated that a similar proportion of primiparas among Croatian primiparas was 46.02% [17]. Won et al. revealed a relatively higher proportion of primiparas (51.6%) in Chinese women [25]. Thrombocytopenia was revealed in 12.1% of patients. The findings of our study are in comparison with those of Ijaz et al. who reported the frequency of thrombocytopenia to be 16.5% among pregnant women [26]. Keihanian et al. also found comparable results of 12.7% in Iranian patients [27], while Onisai et al. reported a rate of 11.1% in Romania [28].

We observed significantly higher frequencies of placental abruption (11.8% vs. 0.0%; p<0.001), preterm delivery (29.4% vs. 0.8%; p<0.001), need for blood transfusion (35.3% vs. 4.5%; p<0.001), stillbirth (8.8% vs. 0.0%; p<0.001), and poor Apgar score (17.6% vs. 0.0%; p<0.001) among pregnant women with thrombocytopenia than those with normal platelet count. Parnas et al. also calculated similar frequencies of placental abruption and preterm delivery as 8.5% and 25.6%, respectively, in pregnant females with thrombocytopenia [10]. The frequency of preterm delivery was presented by Anita et al. in Indian pregnant women with thrombocytopenia, where they reported it to be 31.9% [29]. The need for blood transfusion has been reported by Parnas et al. and Borna et al. as 16.6% and 26.2%, respectively [9,10], while Wyszynski et al. (3.9%) and Parnas et al. (6.5%) reported stillbirth among thrombocytopenic women [10,30]. Parnas et al. also reported a poor Apgar score (16.8%) among newborns of such women [10]. We observed that the frequencies of placental abruption, preterm delivery, need for blood transfusion, stillbirth, and poor Apgar score were all significantly higher than those having a normal platelet count, irrespective of the patient’s age, parity, and BMI. It can be advocated, in light of the results of the present study, that thrombocytopenia is not an uncommon complication during pregnancy and can lead to poor maternal and neonatal outcomes. Being a single-center study, conducted on a relatively modest sample size are the limitations of this study.

## Conclusions

The frequency of thrombocytopenia was found to be 12.1%. The frequency of placental abruption, preterm delivery, need for blood transfusion, stillbirth, and poor Apgar score were all significantly higher among pregnant women with thrombocytopenia as compared to those with a normal platelet count, irrespective of the patient’s age, parity, and BMI. Hence, pregnant women in the third trimester need to be screened for thrombocytopenia, and those positive for it can be taken as high risk so that timely and anticipated management can improve the maternal and neonatal outcomes.
